# Gray matter T1‐w/T2‐w ratios are higher in Alzheimer's disease

**DOI:** 10.1002/hbm.24638

**Published:** 2019-06-03

**Authors:** Wiesje Pelkmans, Ellen Dicks, Frederik Barkhof, Hugo Vrenken, Philip Scheltens, Wiesje M. van der Flier, Betty M. Tijms

**Affiliations:** ^1^ Alzheimer Center Amsterdam, Department of Neurology Amsterdam Neuroscience, Vrije Universiteit Amsterdam, Amsterdam UMC Amsterdam The Netherlands; ^2^ Department of Radiology & Nuclear Medicine Amsterdam Neuroscience, Vrije Universiteit Amsterdam, Amsterdam UMC Amsterdam The Netherlands; ^3^ Institutes of Neurology and Healthcare Engineering, UCL London UK; ^4^ Department of Epidemiology & Biostatistics Amsterdam Neuroscience, Vrije Universiteit Amsterdam, Amsterdam UMC Amsterdam The Netherlands

**Keywords:** Alzheimer's disease, gray matter, myelin content, structural connectivity, T1‐w/T2‐w ratio

## Abstract

Myelin determines the conduction of neuronal signals along axonal connections in networks of the brain. Loss of myelin integrity in neuronal circuits might result in cognitive decline in Alzheimer's disease (AD). Recently, the ratio of T1‐weighted by T2‐weighted MRI has been used as a proxy for myelin content in gray matter of the cortex. With this approach, we investigated whether AD dementia patients show lower cortical myelin content (i.e., a lower T1‐w/T2‐w ratio value). We selected structural T1‐w and T2‐w MR images of 293 AD patients and 172 participants with normal cognition (NC). T1‐w/T2‐w ratios were computed for the whole brain and within 90 automated anatomical labeling atlas regions using SPM12, compared between groups and correlated with the neuronal injury marker tau in cerebrospinal fluid (CSF) and Mini Mental State Examination (MMSE). In contrast to our hypothesis, AD patients showed higher whole brain T1‐w/T2‐w ratios than NC, and regionally in 31 anatomical areas (*p* < .0005; *d* = 0.21 to 0.48), predominantly in the inferior parietal lobule, angular gyrus, anterior cingulate, and precuneus. Regional higher T1‐w/T2‐w values were associated with higher CSF tau concentrations (*p* < .0005; *r* = .16 to .22) and worse MMSE scores (*p* < .0005; *r* = −.16 to −.21). These higher T1‐w/T2‐w values in AD seem to contradict previous pathological findings of demyelination and disconnectivity in AD. Future research should further investigate the biological processes reflected by increases in T1‐w/T2‐w values.

## 1 INTRODUCTION

Alzheimer's disease (AD), the most common cause of dementia, is characterized by a progressive decline in cognition and is one of the major public health challenges of the 21st century (Scheltens et al., 2016). AD is pathologically defined by β‐amyloid (Aβ) neuritic plaques, tau neurofibrillary tangles, and is associated with neuronal, axonal, and synaptic degeneration (Braak & Braak, 1991; Selkoe, 2002). Myelin, the insulating sheath surrounding neuronal axons, is vulnerable to AD pathology while it is essential for efficient neuronal communication by fine‐tuning conduction speed and synchronization, thereby affecting brain connectivity (Nave & Werner, 2014; Timmler & Simons, 2019). A failure in cerebral connectivity has been shown to interfere with healthy cognitive functioning (Fornito, Zalesky, & Breakspear, 2015; Pievani, Filippini, Van Den Heuvel, Cappa, & Frisoni, 2014). In recent years, several proxy measures of myelin content have been proposed, including quantitative magnetic resonance imaging (MRI) techniques such as myelin water fraction (MWF) (Laule et al., 2007) and quantitative susceptibility mapping (QSM) (de Rochefort, Brown, Prince, & Wang, 2008). Moreover, diffusion tensor imaging (DTI) has been used extensively to measure the myelin integrity of large axonal fiber bundles over long distance white matter (WM) networks, which are disrupted in AD (Chua, Wen, Slavin, & Sachdev, 2008; Gao et al., 2014).

An alternative approach of determining regional variation in cortical myelin content, the ratio of the signal intensity of the T1‐weighted (T1‐w) and T2‐weighted (T2‐w) image, has been proposed (Glasser & Van Essen, 2011). The T1‐w/T2‐w technique was developed to parcellate the cerebral cortex from patterns of cortical myelination, and have shown to closely correspond with histological measures of myeloarchitecture (Glasser & Van Essen, 2011; Nieuwenhuys & Broere, 2017). Compared to other quantitative MRI contrasts, this technique has the advantage that images can be acquired during routine clinical MR examinations, with high signal‐to‐noise, and without complex modeling of the MR signal (Heath, Hurley, Johansen‐Berg, & Sampaio‐Baptista, 2018). Along the life‐span, the T1‐w/T2‐w myelin shows an inverted U curve (Grydeland et al., 2019; Grydeland, Walhovd, Tamnes, Westlye, & Fjell, 2013; Shafee, Buckner, & Fischl, 2015), following the maturation of cortical regions throughout life. In addition, higher T1‐w/T2‐w ratios have been associated with greater performance stability during a processing speed task (Grydeland et al., 2013). In disorders with a strong demyelinating component such as multiple sclerosis (MS), the T1‐w/T2‐w ratio is lowered in pathologically vulnerable regions (Beer et al., 2016; Nakamura, Chen, Ontaneda, Fox, & Trapp, 2017). However, recent studies have raised controversy on the interpretation of the T1‐w/T2‐w ratio, showing that this measure may also reflect tissue microstructure other than myelin, such as axon and dendrite density or iron content (Arshad, Stanley, & Raz, 2017; Righart et al., 2017; Uddin, Figley, Marrie, & Figley, 2018; van Rooden et al., 2014). Because disrupted brain connectivity is an important feature of AD (Dicks et al., 2018; Fornito et al., 2015; Pievani et al., 2014), an intracortical reduction of myelin content in AD could be hypothesized. But also other pathological factors associated with AD might lead to intracortical tissue changes. To better understand what this myelin proxy in AD reflects, we compared T1‐w/T2‐w ratio values between older adults with normal cognition and patients with AD‐type dementia for the whole brain and on a regional level. We further investigated the influence of factors that are associated with AD, including cerebrospinal fluid (CSF) total‐tau protein concentration, white matter hyperintensities (WMH), and global cognitive functioning that may contribute to alterations in T1‐w/T2‐w values.

## 2 METHODS

### 2.1 Participants

Individuals with normal cognition (NC) and with AD dementia who had T1‐weighted and T2‐weighted structural MRI were selected from the Amsterdam Dementia Cohort (Van Der Flier et al., 2014; Van Der Flier & Scheltens, 2018). Probable AD dementia was diagnosed according to NIA‐AA criteria with evidence of abnormal levels of Aβ protein (McKhann et al., 2011). Individuals with NC were required to have normal CSF Aβ_1‐42_ levels. Participants were excluded if younger than 40 years old or if the MRI was acquired more than 3 months after clinical diagnosis. All data was collected as part of routine dementia screening.

### 2.2 CSF biomarkers

Collection of CSF by lumbar puncture for all subjects was performed as described previously (Van Der Flier et al., 2014). Aβ_1–42_, phosphorylated tau (p‐tau), and total tau protein concentrations were measured using InnoTest sandwich ELISAs (Innogenetics, Fujirebio, Ghent, Belgium). The cut‐off for abnormal drift‐corrected CSF Aβ_1–42_ was set at <813 ng/L (Tijms et al., 2018), at >375 ng/L for CSF total tau and at >52 ng/L for p‐tau (Mulder et al., 2010).

### 2.3 MRI acquisition

Structural T1‐weighted and T2‐weighted images were acquired as part of routine patient care from a single scanner (3T GE MR750). The following parameters for T1 were used: 3D‐FSPGR, sagittal plane, TR 7.8 ms, TE 3 ms, FA 12°, voxel size 1 mm^3^, 5.06 min; for T2: 2D‐TSE, axial plane, TR 8340 ms, TE 20.7 ms, FA 90°, voxel size 0.5 × 0.5 × 3 mm^3^, 3.35 min. To determine WMH, we additionally analyzed 3D‐fluid attenuation inversion recovery (FLAIR) images, acquired in a sagittal plane, with TR 8,000 ms, TE 126.6 ms, FA 90°, voxel size 1 mm^3^, 5.48 min.

### 2.4 MRI processing

**Figure 1:**
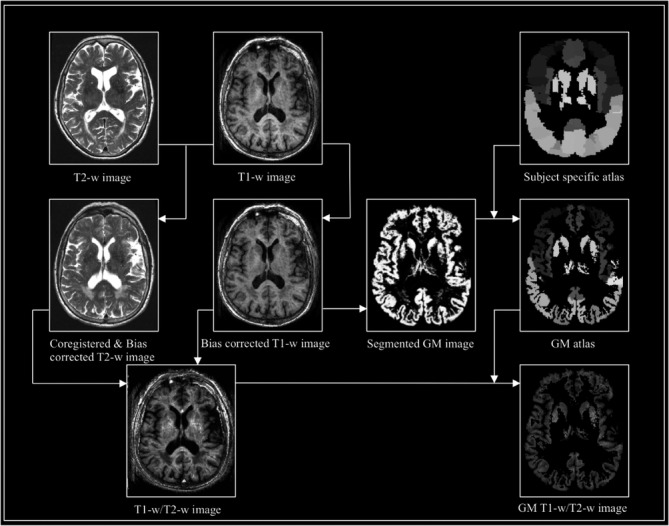
Flow diagram of preprocessing steps to generate T1‐w/T2‐w images. Workflow of T1‐w/T2‐w image data processing using SPM12, including registration of the T2‐w image to the T1w‐w image, bias correction of both images, segmentation, warping of the standard AAL atlas to a subject specific atlas. Finally, the T1‐w/T2‐w image is calculated as the ratio of T1‐w and T2‐w images and masked with a GM mask. AAL, automated anatomical labeling

T1‐w and T2‐w images were converted to Nifti format and analyzed using a similar method as proposed by Glasser and Van Essen (2011). The origin was manually set to the anterior commissure. The T2‐weighted image was rigidly registered with a 4th degree B‐Spline interpolation to the T1‐weighted image using Statistical Parametric Mapping Software version 12 (SPM12; Functional Imaging Laboratory, University College London, London, UK) running in MATLAB 7.12 (MathWorks, Natick, MA). The SPM12 segmentation function allowed for bias field correction of both images, as well as tissue segmentation of the T1‐w images into CSF, GM, and WM tissue probability maps. For validation purposes, we conducted analyses without field bias correction for the T1‐w and T2‐w images as well, obtaining similar results but with less discriminative power to detect differences in intracortical microstructure (Table S2). The data presented here refer to the T1‐w/T2‐w images with bias correction. For each subject, 90 cortical areas were identified with the automated anatomical labeling (AAL) atlas (Tzourio‐Mazoyer et al., 2002), using the SPM normalize function with inverted deformation fields to warp the AAL atlas to Montreal Neurological Institute (MNI) space. Interpolation was set to nearest neighbor. The atlases were masked using subject‐specific masks covering voxels with a GM probability >.3. The T1‐weighted image was then divided by the T2‐weighted image and masked with the GM mask to obtain T1‐w/T2‐w ratios within 90 regions of the AAL atlas (Figure 1). On the FLAIR sequence, WMH were assessed using the Fazekas scale (Fazekas, Chawluk, & Alavi, 1987) (0 = none; 1 = punctuate; 2 = early confluent; and 3 = confluent) and dichotomized into absent (0–1) or present (2–3). All images were visually inspected for segmentation or warping errors; none had to be excluded.

### 2.5 Statistical analysis

Demographic and biomarker data of the diagnostic groups were compared using two‐sample *t* tests for continuous data or chi‐square tests for categorical data. We used general linear models (GLM) to test whether AD patients had globally (i.e., whole cortex) significantly different T1‐w/T2‐w ratios compared to the NC group. This analysis was adjusted for sex and age as the T1‐w/T2‐w ratio has previously shown to vary as a function of age. This model was repeated for regional T1‐w/T2‐w values using Bonferroni correction for multiple comparisons (i.e., *p* value of <.0005 for 90 AAL regions, 0.05/90), and for analysis of individual T1‐w and T2‐w images as well.

To gain further insight into the biological underpinnings of the T1‐w/T2‐w ratio, we further studied the influence of factors known to be associated with AD, that is, WMH, tau, and Mini Mental State Examination (MMSE), on T1‐w/T2‐w values. We used GLM, adjusted for age and sex, to investigate whether WMH severity has an effect on the T1‐w/T2‐w ratio by comparing T1‐w/T2‐w ratio values between individuals with WMH, defined as having a Fazekas score 2–3, to individuals without pronounced WMH (Fazekas 0–1).

Next, we computed a linear regression analysis of CSF total tau concentration and mean T1‐w/T2‐w ratio values for all AAL regions using all subjects and for each group separately. Previous studies have shown that total tau and p‐tau concentrations are highly correlated (Mulder et al., 2010), therefore we chose to analyze the relationship of T1‐w/T2‐w ratios with total‐tau protein only, rather than also p‐tau. Furthermore, the correlation of the whole cortex and regional T1‐w/T2‐w ratio and global cognitive performance score, as measured by the MMSE was calculated. All statistical analyses were performed in R (version 3.5.1), and brain images were visualized with Surf Ice (version 10.14).

## 3 RESULTS

### 3.1 Demographic and clinical data

We included 293 AD and 172 NC subjects. AD patients had higher tau levels than controls, were on average older, more likely to be female, and showed more vascular brain damage, lower total GM volume and total WM volume compared to NC participants (Table 1). The average anatomical distribution of T1‐w/T2‐w values across the cortex in NC participants was qualitatively similar to myelin maps previously reported (Glasser & Van Essen, 2011) (Figure S1). Consistent with their results we observed high T1‐w/T2‐w values in sensory motor regions and visual cortex. The lowest T1‐w/T2‐w values are located in the temporal (pole), anterior cingulate cortex, and frontal areas corresponding with their findings as well.

**Table 1:** Demographic and clinical characteristics according to diagnostic group

	NC	AD	Total
	(*n* = 172)	(*n* = 293)	(*n* = 465)
Sex, F	69 (40%)	159 (54%)*	228 (49%)
Age	59.0 ± 7.3	66.0 ± 7.7*	63.4 ± 8.3
MMSE score	28.3 ± 1.7	20.2 ± 5.0*	23.2 ± 5.7
CSF Aβ_1‐42_	1138.7 ± 178.9	620.9 ± 98.0*	812.5 ± 283.7
CSF t‐tau	229.1 ± 88.4	713.8 ± 466.9*	534.5 ± 441.6
CSF p‐tau	42.8 ± 15.0	90.1 ± 43.1*	72.6 ± 42.2
Vascular damage	11 (6%)	71 (24%)*	82 (18%)
Gray matter volume	659.8 ± 66.9	552.4 ± 63.1*	592.2 ± 82.8
White matter volume	452.3 ± 52.0	415.9 ± 58.2*	429.4 ± 58.6
Cerebrospinal fluid	369.0 ± 78.7	486.3 ± 88.0*	443.0 ± 101.9

*Notes*: Data are presented as mean ± *SD*, or *n* (%). NC, normal cognition; AD, Alzheimer's disease; F, female; age in years; MMSE, Mini Mental State Examination (0–30); CSF, cerebrospinal fluid, CSF in ng/L; vascular damage defined as Fazekas ≥2; volume in mm^3^; (*p < .05).

### 3.2 Altered intracortical T1‐w/T2‐w ratios in AD

**Figure 2:**
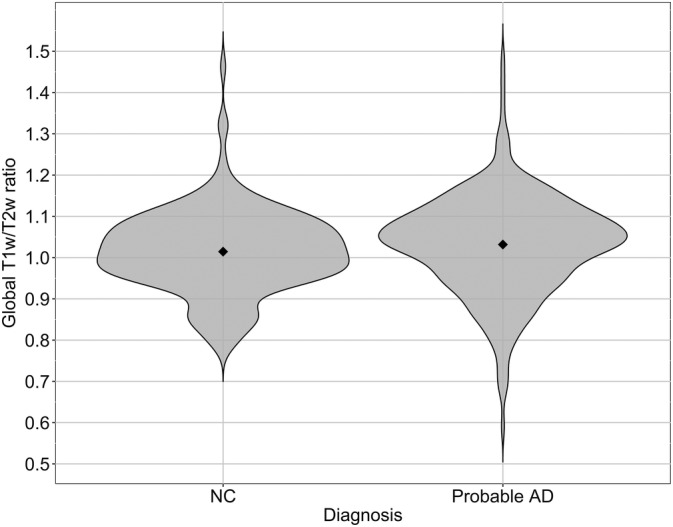
Violin plot representation of mean T1‐w/T2‐w ratio values in all GM regions for AD and NC. Global, that is, whole brain, average T1‐w/T2‐w ratios are significantly higher in AD compared to CN subjects, adjusted for age, and sex. NC (*n* = 172): EMM = 1.004, *SE* = 0.009; AD (*n* = 293): EMM = 1.038, *SE* = 0.007; *F*(1, 464) = 8.326, *d* = 0.15, *p* < .005. Note: plot created with raw data (without covariates). AD, Alzheimer's disease; EMM, estimated marginal means; NC, normal cognition

**Figure 3:**
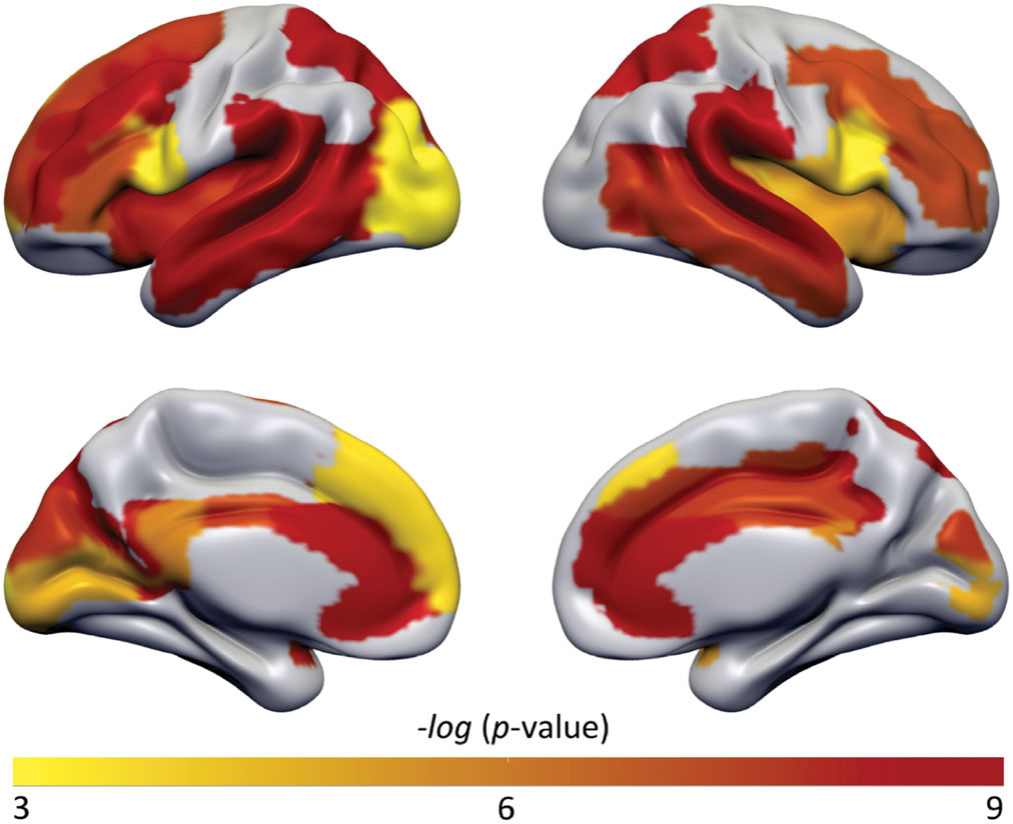
*p*‐value map of AAL regions with significant different T1‐w/T2‐w ratios between AD and NC subjects. Higher T1‐w/T2‐w ratio values were found widespread in AD, in particular in AD‐related regions: superior and middle frontal gyrus; inferior frontal gyrus pars opercularis and triangularis; rolandic operculum; insula; anterior, mid and posterior cingulate gyrus; calcarine; cuneus; middle occipital gyrus; superior and inferior parietal lobule; supramarginal gyrus; angular gyrus; precuneus; superior and middle temporal gyrus. All analyses were adjusted for multiple comparisons showing a moderate (Yellow *p* < .0005) to highly (Red *p* < .000000001) significance scale. AAL, automated anatomical labeling; AD, Alzheimer's disease; NC, normal cognition [Color figure can be viewed at http://wileyonlinelibrary.com]

Average whole cortex T1‐w/T2‐w values were higher in AD compared to NC [NC: EMM = 1.004, *SE* = 0.009; AD: EMM = 1.038, *SE* = 0.007; *F*(1, 464) = 8.33, *d* = 0.15, *p* < .01] (Estimated marginal means (EMM); Figure 2). At regional level stronger effects were found, with multiple brain regions showing higher T1‐w/T2‐w values in AD (Figure 3). The largest differences were observed in the anterior cingulate, inferior parietal lobule, supramarginal gyrus, angular gyrus, precuneus, and superior temporal gyrus (range *d* = 0.21 to *d* = 0.48; *p* < .0005 to *p* < .00000001; Table S1). No regions with significantly lower values were observed in AD.

**Figure 4:**
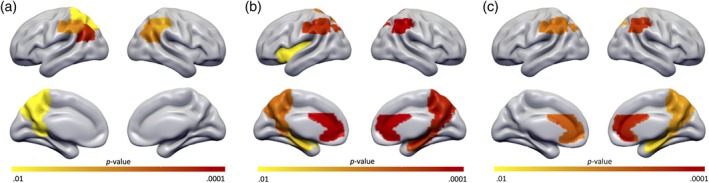
*p*‐value map of AAL regions with different T1‐w images, T2‐w images, and 1/T2‐w images between AD and NC subjects. Figures shown are uncorrected for multiple comparisons to give insight into the pattern of regions that differed between groups at a trend level. (a) Surfaceplot shows a trend of higher T1‐w image intensity in AD patients in the inferior parietal lobule, angular gyrus, precuneus, and superior parietal lobule. (b) T2‐w images had significantly lower intensities in the anterior cingulate in the AD group. And showed a trend toward lower T2‐w image intensity in AD patients in the inferior parietal lobule, precuneus, hippocampus, and insula. (c) Surfaceplot shows a trend of higher 1/T2‐w image intensity in AD patients in the anterior cingulate gyrus, inferior parietal lobule, hippocampus, and precuneus. Displayed are regions with a *p*‐value scale of (yellow *p* < .01) to (red *p* < .0001). AAL, automated anatomical labeling; AD, Alzheimer's disease; NC, normal cognition [Color figure can be viewed at http://wileyonlinelibrary.com]

Comparisons of individual images between diagnostic groups resulted in no significant differences in T1‐w image intensity. However uncorrected results showed a trend of higher T1‐w image intensity in AD patients in the inferior parietal lobule, angular gyrus, precuneus, and superior parietal lobule. T2‐w images showed significantly lower intensities in the anterior cingulate in the AD group compared to NC (*p* < .0005; Figure 4). And a trend toward lower T2‐w image intensity in AD patients in the inferior parietal lobule, precuneus, hippocampus, and insula. Moreover, to exclude possible age or sex effects, the main analysis was rerun using a subset (*n* = 50) of age‐ and sex‐matched individuals. Obtaining similar results, albeit less significant (Table S3).

### 3.3 Association of T1‐w/T2‐w ratio values with other AD markers

**Figure 5:**
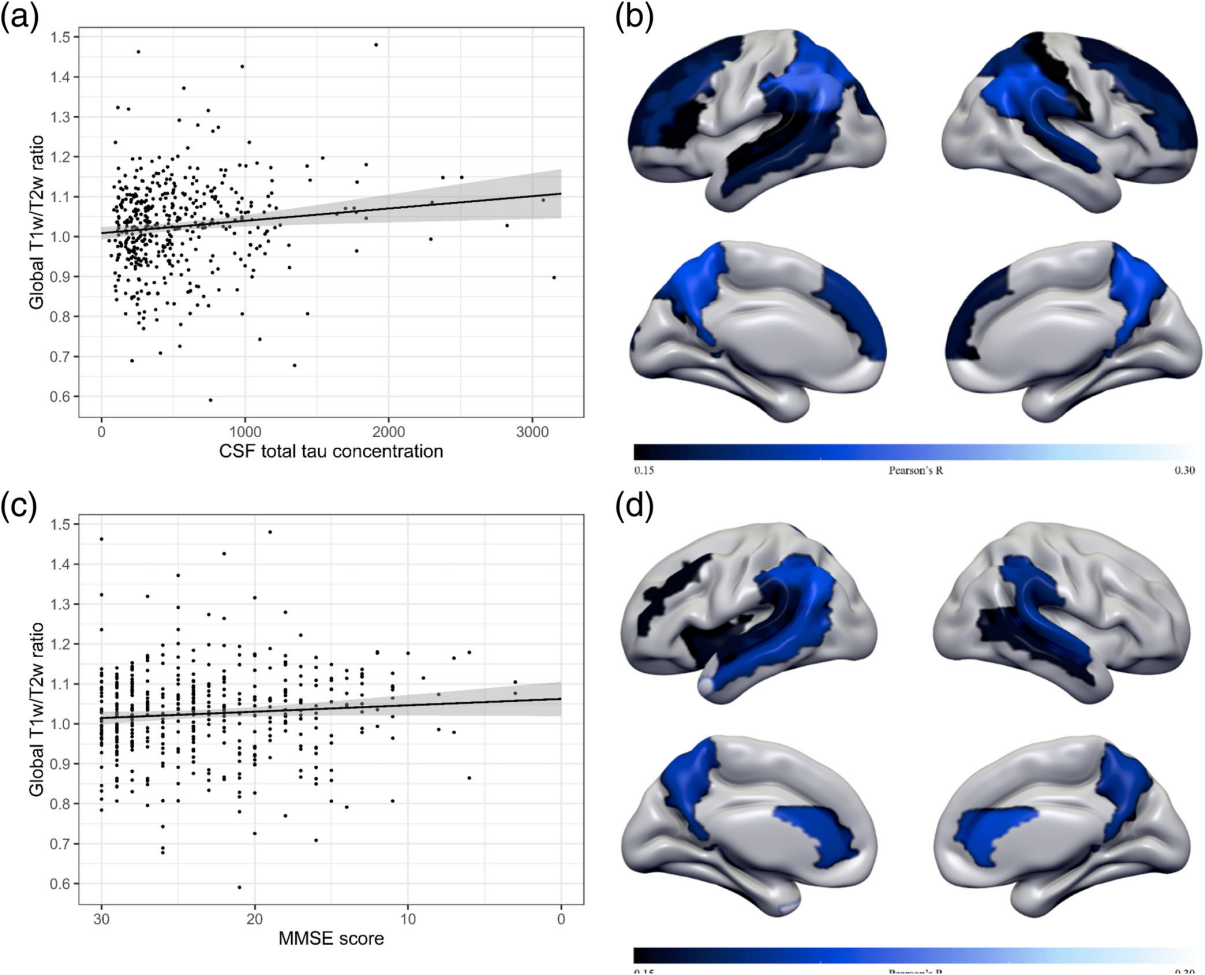
Association of T1‐w/T2‐w ratio values with other AD markers. (a) Global T1‐w/T2‐w ratio as a function of tau. Scatterplot showing an association of higher T1‐w/T2‐w ratios with abnormal CSF tau concentrations in the total sample. Regression line in black, the 95% CI in shaded gray. (b) Surfacemap showing regional associations of CSF total tau concentration with T1‐w/T2‐w values. Higher tau concentrations are associated with higher T1‐w/T2‐w ratios in the superior and middle frontal gyrus, pars triangularis, medial frontal gyrus, superior occipital, postcentral gyrus, superior and inferior parietal lobule, supramarginal gyrus, angular gyrus, precuneus, and superior temporal gyrus across all subjects (range *r* = .15 to *r* = .22; *p* = .0005 to *p* = .000001). (c) Global T1‐w/T2‐w ratio as a function of MMSE score. Scatterplot showing the relation between global T1‐w/T2‐w ratios according to MMSE score in the total sample. Regression line in black, the 95% CI in shaded gray. (d) Surfacemap showing regional associations of MMSE scores with T1‐w/T2‐w values. Lower MMSE scores are associated with higher T1‐w/T2‐w ratios in the middle frontal gyrus, insula, anterior cingulate gyrus, inferior parietal lobule, supramarginal gyrus, angular gyrus, precuneus, superior and middle temporal gyrus across all subjects (range *r* = .15 to *r* = .21; *p* = .0005 to *p* = .000008). AD, Alzheimer's disease; CSF, cerebrospinal fluid; MMSE, Mini Mental State Examination [Color figure can be viewed at http://wileyonlinelibrary.com]

There was no significant difference between the group with WMH and the group without WMH in T1‐w/T2‐w values, regional or whole brain [*F*(1, 463) = 0.92, *p* = .34, *d* = 0.004]. Next, the effect of the neuronal injury marker tau on the mean global T1‐w/T2‐w ratio was explored. The regression analysis indicated that T1‐w/T2‐w ratios across the whole cortex increased with higher tau concentrations in CSF (*r* = .11, *p* < .01; Figure 5a). These associations were specific for the AD group (AD: *r* = .12, *p* < .05; CN: *r* = −.07, *p* = .40) and located in mostly frontal and parietal regions with significant correlation values ranging from *r* = .16 to .22 (all *p* < .0005; Figure 5b), and remained when adjusting for age and sex. Note that we did not investigate the relationship of CSF Aβ levels and T1‐w/T2‐w ratio as the diagnostic groups already reflect this association, because CN and AD subjects were selected based on their amyloid status. We further analyzed the mean whole cortex correlation between T1‐w/T2‐w ratio and MMSE scores which was *r* = −.08, with *p*‐value = .08 (AD: *r* = −.05, *p* = .34; CN: *r* = .08, *p* = .34). Which implies that worse global cognitive functioning correlated with higher T1‐w/T2‐w ratios at a trend level (Figure 5c), and regionally stronger associations were observed, which remained when adjusting for age and sex, mostly in temporal, cingulate, and parietal brain areas (range *r* = −.16 to *r* = −.21, all *p* < .0005; Figure 5d).

## 4 DISCUSSION

The main finding of our study was that T1‐w/T2‐w ratio values were higher in AD compared to controls, which was contrary to our expectation. These changes tended to be most pronounced in anatomical areas known to be affected in AD such as the interior parietal lobule and precuneus, and were associated with higher levels of the neuronal injury marker tau and worse cognition.

Previous studies have suggested that myelin is the predominant source of MR contrast in T1‐weighted and T2‐weighted images (Bock, Kocharyan, Liu, & Silva, 2009; Eickhoff et al., 2005; Laule et al., 2007; Wallace et al., 2016), and by using the ratio of these images the contrast sensitivity to detect myelin content is increased further due to attenuation of the shared intensity biases (Glasser & Van Essen, 2011). Although, other studies have questioned the sensitivity of the T1‐w/T2‐w ratio for myelin content, and argued that it is likely not the only factor contributing to the T1‐w/T2‐w image (Arshad et al., 2017; Righart et al., 2017; Uddin et al., 2018). Still, it has been shown that T1‐w/T2‐w ratio differentiates between high and low myelinated cortex as determined by myelin proteolipid protein staining (Nakamura et al., 2017). Also in our study, we observed no indication that our T1‐w/T2‐w map differed from those previously reported in NC, with high T1‐w/T2‐w values in regions such as the basal ganglia, thalamus, and paracentral lobule (Ganzetti, Wenderoth, & Mantini, 2015) and low T1‐w/T2‐w ratios in the insula, anterior cingulate, hippocampus, medial orbitofrontal gyrus, and temporal pole (Fischl et al., 2004; Glasser, Goyal, Preuss, Raichle, & Van Essen, 2014), suggesting it correlates well with cortical myelination.

To the best of our knowledge, this technique has not been applied in AD dementia before, however, a previous study showed that higher T1‐w/T2‐w values correlated with amyloid beta deposition on positron‐emission tomography (PET) in individuals with normal cognition (Yasuno et al., 2017). Our observation of higher T1‐w/T2‐w seems in line with that study. The authors found higher regional T1‐w/T2‐w ratios in the frontal cortex and anterior cingulate in subjects with high PiB binding compared to subjects with low PiB binding and suggested that microstructural alterations induced by amyloid beta could be detected with the T1‐w/T2‐w ratio. In our study, the higher T1‐w/T2‐w ratio in AD also seem to be regionally specific, showing the highest T1‐w/T2‐w ratios in AD patients in the angular gyrus, inferior parietal lobule, precuneus, anterior cingulate, supramarginal gyrus, and superior temporal gyrus. These high T1‐w/T2‐w regions overlap with areas known be involved in AD, and have been associated with the “default mode network,” a set of brain areas that shows high functional correlations when a person is not performing a task. In particular, these regions seem most vulnerable for early amyloid depositions and decreased functional connectivity in AD (Buckner, 2005; Palmqvist et al., 2017). Additionally, this corresponds with numerous studies showing that particularly late‐myelinating, that is temporal and frontal, intracortical fibers seem particularly prone to lose myelin (Bartzokis et al., 2004; Brettschneider, Del Tredici, Lee, & Trojanowski, 2015; Jucker & Walker, 2018; Thal, Rüb, Orantes, & Braak, 2002). The sensory and motor regions, that is early myelinating, showed little difference in T1‐w/T2‐w ratio between AD and NC individuals. These regions are known to have a relative sparing of AD pathology until late disease stages (Bartzokis, Lu, & Mintz, 2007). Although the relationships were not strong, our findings showed an association of higher T1‐w/T2‐w ratios in mostly frontal and parietal areas with increased tau pathology, and higher T1‐w/T2‐w ratios in mainly temporal areas with a decline in global cognitive functioning, suggesting that the cortical changes detected by the T1‐w/T2‐w ratio show a regional vulnerability and an association with pathology.

Additionally, we analyzed the intensity measures derived from the T1‐w and T2‐w images separately. GM T1‐w images did not show significant differences in intensity between NC and AD groups. However, T2‐w images had significantly lower intensities in the anterior cingulate and inferior parietal lobule in the AD group compared to NC. Therefore, the group difference in T1‐w/T2‐w ratios seems to be predominantly driven by the overall lower T2‐w signal in AD patients. A finding that might be related to increased Aβ protein concentrations, which are known to decrease the T2‐w signal (Imon et al., 1995). When applying the ratio of T1‐w by T2‐w images, it greatly improves the sensitivity to detect subtle differences, than using single modalities separately.

However, it remains unclear what biological substrate higher T1‐w/T2‐w values reflect. Disorders with a strong demyelinating component such as MS, have consistently showed lower T1‐w/T2‐w values in pathologically vulnerable regions (Beer et al., 2016; Nakamura et al., 2017; Righart et al., 2017). Additionally, lowered T1‐w/T2‐w ratios in several brain regions in schizophrenia (Ganzetti et al., 2015; Iwatani et al., 2015) and bipolar disorder patients (Ishida et al., 2017) have been identified. Recent transcriptomic studies have further observed correlations of the T1‐w/T2‐w intensity in the cortex with expression of genes associated with cortical microcircuit specialization and myelin (Burt et al., 2018; Ritchie, Pantazatos, & French, 2018). In contrast, recently a study with Parkinson disease patients showed a higher T1‐w/T2‐w ratio in the substantia nigra pars compacta compared to controls (Du, Lewis, Sica, Kong, & Huang, 2018). Also in Huntington disease, higher T1‐w/T2‐w ratios in several cortical regions including the insula, ventrolateral frontal cortex, and medial temporal pole were found (Rowley et al., 2018). This incongruency suggests that in AD either demyelination does not occur, or the T1‐w/T2‐w ratio measures something different rather than myelin content. Still, structural imaging and histological studies have reported a disruption in WM integrity in AD (Bartzokis et al., 2004; Carmichael et al., 2010; Filley & Fields, 2016; Nir et al., 2013; Rowley et al., 2013). For example, more white matter hyperintensities have been related to an increased risk of dementia and cognitive deficits, independently of vascular risk factors and stroke in the AD continuum (Debette et al., 2010; Gordon et al., 2015; Nir et al., 2013). Cortical demyelination in the context of AD has been demonstrated also in the medial temporal lobe and lingual gyri using the magnetization transfer ratio in mild cognitive impairment patients (Carmeli et al., 2013). Moreover, focal demyelination of the cortical gray matter has been reported around Aβ plaques, suggesting a vulnerability of myelin to Aβ toxicity (Mitew et al., 2010). Therefore, we consider it unlikely that no demyelination occurs in AD. Another possibility is remyelination (Peters, 2009), which has also been suggested by a recent study by Bulk et al. (2018). Using a combined post mortem T2*w MRI with histology, the authors showed an unexpected increase in cortical myelin staining in late stage AD (Bulk et al., 2018). The increase in myelin density did show a more disorganized cortical myelin architecture compared to controls. Furthermore, Van Duijn et al. (2017) also found increased myelin protein labeling in regions affected severely by Aβ and tau pathology. Possibly, adequate intracortical myelin plasticity may initially compensate for the subcortical transmission delays along WM subcortical fibers, but eventually during the disease course significant intracortical oligodendrocyte deficits develop. Note however, that Bulk et al. (2018), and multiple other studies (Ayton et al., 2019; Connor, Snyder, Beard, Fine, & Mufson, 1992; Ward, Zucca, Duyn, Crichton, & Zecca, 2014; Zecca, Youdim, Riederer, Connor, & Crichton, 2004), also demonstrated increased iron accumulation in AD. T2‐w sequences are known to be highly sensitive to iron deposits, and so the T1‐w/T2‐w ratio may reflect the presence of both processes (Dusek, Dezortova, & Wuerfel, 2013). Because there is a strong colocalization of myelin and iron in the cortex (Fukunaga et al., 2010; Quintana et al., 2006; Stüber et al., 2014), this would imply that even though iron may contribute to the T1‐w/T2‐w signal, the T1‐w/T2‐w signal would still mainly (directly and indirectly) reflect the underlying myeloarchitecture. However in pathological conditions such as AD, the relationship of myelin and iron may be altered, for instance by loss of oligodendrocytes or iron rich depositions around Aβ plaques (Hare, Ayton, Bush, & Lei, 2013). There is also evidence that brain regions originally low in iron content, such as the frontal and temporal lobe, appear to be most vulnerable to iron dyshomeostasis and are susceptible to amyloid pathology (Connor et al., 1992; Hallgren & Sourander, 1958). In other words, there appears to be a synergy of iron and β‐amyloid toxicity in specific brain regions that overlap with regions we observed to show high T1‐w/T2‐w ratios in AD. Future research should further investigate this question using a combined PET and MR approach. To summarize, the T1‐w/T2‐w ratio is likely to be influenced by other microstructural factors than myelin and does not seem suited as a measure for disrupted cortical myelination in diseased cohorts. Rather, more studies are needed to measure these other pathological processes at the same time in AD to further understand what T1‐w/T2‐w changes in AD reflect.

There are some limitations to our study. As data was collected in clinical setting, our analysis were not identical to the original approach of Glasser and Van Essen (2011), including different acquisition parameters, that is, isotropic voxels for both images versus nonisotropic voxels for T2‐w images in our study. As a consequence, our analyses may be more prone to partial volume effects. To study possible contamination from non‐GM tissue classes, we repeated analyses in a subset of individuals including only voxels with increasing GM probabilities and the use of an eroded GM mask. Findings were consistent to the main analyses, suggesting that our results cannot simply be explained by differences in partial volume effects (Figures S2 and S3). Furthermore, as we used a volumetric approach, we are unable to exclude the possibility that cortical layering patterns may have influenced the T1‐w/T2‐w ratio. Future studies should compare volumetric approaches with for example, a surface‐based registration that might better account for each person's cortical folding pattern (Fischl, Sereno, & Dale, 1999). Moreover, the differences in T1‐w/T2‐w ratio between AD and NC individuals are highly significant, however the effect sizes are too small to be of clinical significance. Another limitation is that we have no pathological data available to further study whether T1‐w/T2‐w corresponds to myelin in the same individuals: future research should take confounding effects such as iron and inflammation into account and histologically quantify the T1‐w/T2‐w in healthy and pathological tissue. Alongside this, the large sample size, the use of the same scanning protocol for all subjects, and the use of AD biomarkers strengthens our study.

In conclusion, our findings of higher T1‐w/T2‐w values in AD suggest that this measure may not (only) reflect myelin content. Currently the T1‐w/T2‐w ratio is used frequently across various populations to characterize myelin. Our results suggest that this measure should be interpreted with caution in particular in disease populations and further validation and characterization of the T1‐w/T2‐w ratio and its neurobiological origin in AD is necessary for correct interpretation.

## DISCLOSURES

W. Pelkmans and E. Dicks report no disclosures. F. Barkhof is a consultant for Biogen‐Idec, Janssen Alzheimer Immunotherapy, Bayer‐Schering, Merck‐Serono, Roche, Novartis, Genzume, and Sanofi‐Aventis; has received sponsoring from European Commission–Horizon 2020, National Institute for Health Research–University College London Hospitals Biomedical Research Centre, Scottish Multiple Sclerosis Register, TEVA, Novartis, and Toshiba; is supported by the University College London Hospitals NHS Foundation Trust Biomedical Research Center; and serves on the editorial boards of Radiology, Brain, Neuroradiology, Multiple Sclerosis Journal, and Neurology. H. Vrenken reports no disclosures. P. Scheltens has acquired grant support (for the institution) from BiogenGE Healthcare, Danone Research, Piramal, and Merck. In the past 2 years, he has received consultancy/speaker fees (paid to the institution) from Lilly, GE Healthcare, Novartis, Sanofi, Nutricia, Probiodrug, Biogen, Roche, Avraham, and EIP Pharma, Merck AG. W. M. van der Flier's research programs have been funded by ZonMW, the Netherlands Organization of Scientific Research, Seventh European Framework Programme, Alzheimer Nederland, Cardiovascular Onderzoek Nederland, Stichting Dioraphte, Gieskes‐Strijbis fonds, Boehringer Ingelheim, Piramal Imaging, Roche BV, Janssen Stellar, Biogen MA and Combinostics. All funding is paid to her institution. B. M. Tijms declares that she is supported by the ZonMW Memorabel “(Grant number #73305056).”

## Supporting information


**Supplementary figure 1 Whole brain group average T1‐w/T2‐w ratio surfacemap of subjects with normal cognition.** The signal strength distribution shows relatively high T1‐w/T2‐w values (myelin content) in sensory and motor regions and lower values in temporal and frontal areas corresponding with previous literature.

**Supplementary figure 2 Scatterplot of global T1‐w/T2‐w ratios with CSF and WM volume using different GM probabilities.** In the main analysis the T1‐w/T2‐w image was masked using a mask with a GM probability of >.3. In a subset of N = 50 (25 NC; 25 AD) subjects, data was reanalyzed using a GM mask probability of >.1 and > .9. These T1‐w/T2‐w values were correlated with CSF and WM volumes. The results of both images with different GM probabilities are very similar, and no increase in T1‐w/T2‐w ratios was seen with higher CSF or WM volume. Therefore, we think it is unlikely that our main results, that is, higher T1‐w/T2‐w ratios in AD, can be fully explained by partial volume effects.

**Supplementary figure 3 Scatterplot of global T1‐w/T2‐w ratios masked with an eroded GM mask with CSF and WM volume.** In a subset of N = 50 (25 NC; 25 AD), the data was re‐run using an eroded GM mask to reduce possible partial volume effects from neighboring GM voxels. Using the erode function in fslmaths, the subject specific GM masks were eroded when zero voxels was found in kernel. The eroded GM mask actually resulted in more PVE, likely associated with atrophy and was therefore not applied in the main analysis.

**Supplementary table 1 Table of mean T1‐w/T2‐w ratio values for all AAL brain regions compared between AD and NC subjects.** Areas showing significantly higher T1‐w/T2‐w ratios in AD compared to CN subjects are displayed in bold.
**Supplementary Table 2. Table of mean T1‐w/T2‐w ratio values for all AAL brain regions compared between AD and NC subjects without image bias correction.** Areas showing significantly higher T1‐w/T2‐w ratios in AD compared to CN subjects are displayed in bold.
**Supplementary Table 3. Table of mean T1‐w/T2‐w ratio values for all AAL brain regions compared between AD and NC subjects using age and sex matched individuals (n = 50).** Areas showing significantly higher T1‐w/T2‐w ratios in AD compared to CN subjects are displayed in bold.
