# Supplementary material for: Gray matter T1‐w/T2‐w ratios are higher in Alzheimer's disease
Source: Hum Brain Mapp. 2019 Jun 3;40(13):3900–9. doi: 10.1002/hbm.24638 (PMC6771703; doi:10.1002/hbm.24638)
Supplement: Supplementary file 4 — Supplementary table 1 Table of mean T1‐w/T2‐w ratio values for all AAL brain regions compared between AD and NC subjects. Areas showing significantly higher T1‐w/T2‐w ratios in AD compared to CN subjects are displayed in bold. Supplementary Table 2. Table of mean T1‐w/T2‐w ratio values for all AAL brain regions compared between AD and NC subjects without image bias correction. Areas showing significantly higher T1‐w/T2‐w ratios in AD compared to CN subjects are displayed in bold. Supplementary Table 3. Table of mean T1‐w/T2‐w ratio values for all AAL brain regions compared between AD and NC subjects using age and sex matched individuals (n = 50). Areas showing significantly higher T1‐w/T2‐w ratios in AD compared to CN subjects are displayed in bold. [file HBM-40-3900-s004.docx]

Supplementary information that accompanies this paper:

**Supplementary table 1.** **Table of mean T1-w/T2-w ratio values for all AAL brain regions compared between AD and NC subjects.** Areas showing significantly higher T1-w/T2-w ratios in AD compared to CN subjects are displayed in bold.

|  |  | |  |  |  |  |  |  |
| --- | --- | --- | --- | --- | --- | --- | --- | --- |
| **AAL** | | **Region** | **Diagnosis** | ***EMM*** | ***SE*** | ***F*** | ***d*** | **P-value** |
| 1 | Precentral Gyrus L | | NC | 1.08 | .010 | 6.26 | .10 | .01266790 |
|  |  | | AD | 1.12 | .007 |  |  |  |
| 2 | Precentral Gyrus R | | NC | 1.12 | .010 | 3.25 | .03 | .07174438 |
|  |  | | AD | 1.14 | .008 |  |  |  |
| 3 | Superior Frontal Gyrus L | | NC | 1.00 | .009 | 15.07 | .26 | **.00011849** |
|  |  | | AD | 1.05 | .007 |  |  |  |
| 4 | Superior Frontal Gyrus R | | NC | 1.01 | .009 | 11.41 | .21 | .00079088 |
|  |  | | AD | 1.05 | .007 |  |  |  |
| 5 | Superior Orbital Frontal Gyrus L | | NC | .98 | .008 | 7.60 | .17 | .00607814 |
|  |  | | AD | 1.01 | .006 |  |  |  |
| 6 | Superior Orbital Frontal Gyrus R | | NC | .98 | .008 | 6.95 | .16 | .00868921 |
|  |  | | AD | 1.01 | .006 |  |  |  |
| 7 | Middle Frontal Gyrus L | | NC | .98 | .009 | 18.36 | .35 | **.00002227** |
|  |  | | AD | 1.03 | .007 |  |  |  |
| 8 | Middle Frontal Gyrus R | | NC | .99 | .009 | 14.64 | .30 | **.00014814** |
|  |  | | AD | 1.04 | .007 |  |  |  |
| 9 | Middle Orbital Frontal Gyrus L | | NC | .95 | .008 | 8.46 | .20 | .00380910 |
|  |  | | AD | .98 | .006 |  |  |  |
| 10 | Middle Orbital Frontal Gyrus R | | NC | .96 | .008 | 5.56 | .13 | .01882445 |
|  |  | | AD | .99 | .006 |  |  |  |
| 11 | Inferior Frontal Gyrus Pars Opercularis L | | NC | .97 | .009 | 12.37 | .26 | **.00047944** |
|  |  | | AD | 1.01 | .007 |  |  |  |
| 12 | Inferior Frontal Gyrus Pars Opercularis R | | NC | .97 | .009 | 12.47 | .25 | **.00045408** |
|  |  | | AD | 1.01 | .007 |  |  |  |
| 13 | Inferior Frontal Gyrus Pars Triangularis L | | NC | .96 | .009 | 14.39 | .30 | **.00016812** |
|  |  | | AD | 1.01 | .007 |  |  |  |
| 14 | Inferior Frontal Gyrus Pars Triangularis R | | NC | .98 | .009 | 11.53 | .26 | .00074575 |
|  |  | | AD | 1.02 | .007 |  |  |  |
| 15 | Inferior Frontal Gyrus Pars Orbitalis L | | NC | 1.00 | .009 | 7.50 | .17 | .00642322 |
|  |  | | AD | 1.03 | .006 |  |  |  |
| 16 | Inferior Frontal Gyrus Pars Orbitalis R | | NC | 1.03 | .009 | 4.40 | .12 | .03640697 |
|  |  | | AD | 1.05 | .007 |  |  |  |
| 17 | Rolandic Operculum L | | NC | .99 | .010 | 10.58 | .22 | .00122432 |
|  |  | | AD | 1.03 | .007 |  |  |  |
| 18 | Rolandic Operculum R | | NC | .96 | .009 | 12.90 | .24 | **.00036377** |
|  |  | | AD | 1.01 | .007 |  |  |  |
| 19 | Supplementary Motor Area L | | NC | 1.03 | .010 | 4.86 | .07 | .02803077 |
|  |  | | AD | 1.06 | .007 |  |  |  |
| 20 | Supplementary Motor Area R | | NC | 1.03 | .010 | 5.85 | .08 | .01599272 |
|  |  | | AD | 1.05 | .007 |  |  |  |
| 21 | Olfactory Cortex L | | NC | .93 | .009 | 5.63 | .10 | .01810567 |
|  |  | | AD | .96 | .006 |  |  |  |
| 22 | Olfactory Cortex R | | NC | .93 | .008 | 5.80 | .13 | .01643551 |
|  |  | | AD | .95 | .006 |  |  |  |
| 23 | Medial Frontal Gyrus L | | NC | .96 | .009 | 12.62 | .29 | **.00042139** |
|  |  | | AD | 1.00 | .007 |  |  |  |
| 24 | Medial Frontal Gyrus R | | NC | .95 | .009 | 10.22 | .24 | .00148292 |
|  |  | | AD | .99 | .007 |  |  |  |
| 25 | Medial Orbitofrontal Cortex L | | NC | .91 | .008 | 7.16 | .19 | .00773805 |
|  |  | | AD | .93 | .006 |  |  |  |
| 26 | Medial Orbitofrontal Cortex R | | NC | .88 | .008 | 8.04 | .21 | .00476612 |
|  |  | | AD | .91 | .006 |  |  |  |
| 27 | Gyrus Rectus L | | NC | .93 | .008 | 4.28 | .12 | .03907625 |
|  |  | | AD | .95 | .006 |  |  |  |
| 28 | Gyrus Rectus R | | NC | .92 | .008 | 4.75 | .10 | .02981004 |
|  |  | | AD | .95 | .006 |  |  |  |
| 29 | Insula L | | NC | .85 | .008 | 15.72 | .34 | **.00008521** |
|  |  | | AD | .89 | .006 |  |  |  |
| 30 | Insula R | | NC | .87 | .008 | 13.07 | .29 | **.00033322** |
|  |  | | AD | .91 | .006 |  |  |  |
| 31 | Anterior Cingulate Gyrus L | | NC | .87 | .009 | 19.43 | .46 | **.00001298** |
|  |  | | AD | .92 | .006 |  |  |  |
| 32 | Anterior Cingulate Gyrus R | | NC | .86 | .009 | 23.57 | .48 | **.00000165** |
|  |  | | AD | .91 | .006 |  |  |  |
| 33 | Midcingulate Area L | | NC | .92 | .009 | 10.50 | .26 | .00128381 |
|  |  | | AD | .96 | .007 |  |  |  |
| 34 | Midcingulate Area R | | NC | .89 | .009 | 15.07 | .33 | **.00011846** |
|  |  | | AD | .94 | .006 |  |  |  |
| 35 | Posterior Cingulate Cyrus L | | NC | 1.04 | .010 | 13.52 | .25 | **.00026328** |
|  |  | | AD | 1.08 | .007 |  |  |  |
| 36 | Posterior Cingulate Cyrus R | | NC | 1.05 | .009 | 3.64 | .04 | .05690315 |
|  |  | | AD | 1.07 | .007 |  |  |  |
| 37 | Hippocampus L | | NC | .87 | .008 | 1.58 | .32 | .20915277 |
|  |  | | AD | .86 | .006 |  |  |  |
| 38 | Hippocampus R | | NC | .90 | .008 | 4.07 | .41 | .04412484 |
|  |  | | AD | .88 | .006 |  |  |  |
| 39 | Parahippocampal Gyrus L | | NC | .96 | .009 | .10 | .16 | .75176180 |
|  |  | | AD | .97 | .006 |  |  |  |
| 40 | Parahippocampal Gyrus R | | NC | .96 | .009 | .00 | .19 | .94944799 |
|  |  | | AD | .96 | .006 |  |  |  |
| 41 | Amygdala L | | NC | .98 | .009 | 1.27 | .07 | .25958119 |
|  |  | | AD | .99 | .007 |  |  |  |
| 42 | Amygdala R | | NC | .98 | .009 | 1.82 | .04 | .17862583 |
|  |  | | AD | 1.00 | .007 |  |  |  |
| 43 | Calcarine Sulcus L | | NC | 1.10 | .010 | 12.97 | .21 | **.00035107** |
|  |  | | AD | 1.15 | .007 |  |  |  |
| 44 | Calcarine Sulcus R | | NC | 1.12 | .010 | 9.86 | .14 | .00179885 |
|  |  | | AD | 1.16 | .008 |  |  |  |
| 45 | Cuneus L | | NC | 1.04 | .009 | 15.21 | .23 | **.00011040** |
|  |  | | AD | 1.09 | .007 |  |  |  |
| 46 | Cuneus R | | NC | 1.08 | .010 | 9.76 | .14 | .00190088 |
|  |  | | AD | 1.12 | .007 |  |  |  |
| 47 | Lingual Gyrus L | | NC | 1.08 | .010 | 5.15 | .08 | .02372393 |
|  |  | | AD | 1.11 | .007 |  |  |  |
| 48 | Lingual Gyrus R | | NC | 1.11 | .010 | 1.97 | .01 | .16167314 |
|  |  | | AD | 1.13 | .008 |  |  |  |
| 49 | Superior Occipital Gyrus L | | NC | 1.02 | .010 | 10.02 | .17 | .00165187 |
|  |  | | AD | 1.06 | .007 |  |  |  |
| 50 | Superior Occipital Gyrus R | | NC | 1.04 | .010 | 5.85 | .10 | .01596634 |
|  |  | | AD | 1.07 | .007 |  |  |  |
| 51 | Middle Occipital Gyrus L | | NC | .99 | .009 | 12.16 | .22 | .00053444 |
|  |  | | AD | 1.03 | .007 |  |  |  |
| 52 | Middle Occipital Gyrus R | | NC | .98 | .009 | 8.69 | .15 | .00335976 |
|  |  | | AD | 1.02 | .007 |  |  |  |
| 53 | Inferior Occipital Cortex L | | NC | 1.01 | .009 | 4.94 | .06 | .02666731 |
|  |  | | AD | 1.04 | .007 |  |  |  |
| 54 | Inferior Occipital Cortex R | | NC | 1.04 | .009 | .89 | .06 | .34534276 |
|  |  | | AD | 1.06 | .007 |  |  |  |
| 55 | Fusiform Gyrus L | | NC | .96 | .009 | 5.26 | .07 | .02231508 |
|  |  | | AD | .99 | .007 |  |  |  |
| 56 | Fusiform Gyrus R | | NC | .96 | .009 | 2.30 | .01 | .13050923 |
|  |  | | AD | .98 | .006 |  |  |  |
| 57 | Postcentral Gyrus L | | NC | 1.09 | .010 | 8.87 | .17 | .00305913 |
|  |  | | AD | 1.13 | .008 |  |  |  |
| 58 | Postcentral Gyrus R | | NC | 1.08 | .010 | 9.52 | .17 | .00216084 |
|  |  | | AD | 1.12 | .008 |  |  |  |
| 59 | Superior Parietal Lobule L | | NC | 1.05 | .010 | 20.34 | .30 | **.00000826** |
|  |  | | AD | 1.11 | .008 |  |  |  |
| 60 | Superior Parietal Lobule R | | NC | 1.05 | .010 | 16.01 | .26 | **.00007337** |
|  |  | | AD | 1.10 | .008 |  |  |  |
| 61 | Inferior Parietal Lobule L | | NC | 1.03 | .010 | 32.34 | .44 | **.00000002** |
|  |  | | AD | 1.11 | .008 |  |  |  |
| 62 | Inferior Parietal Lobule R | | NC | 1.02 | .010 | 33.61 | .43 | **.00000001** |
|  |  | | AD | 1.10 | .007 |  |  |  |
| 63 | Supramarginal Gyrus L | | NC | .95 | .009 | 21.73 | .32 | **.00000412** |
|  |  | | AD | 1.00 | .007 |  |  |  |
| 64 | Supramarginal Gyrus R | | NC | .96 | .009 | 23.67 | .36 | **.00000157** |
|  |  | | AD | 1.02 | .007 |  |  |  |
| 65 | Angular Gyrus L | | NC | .95 | .009 | 33.67 | .45 | **.00000001** |
|  |  | | AD | 1.02 | .007 |  |  |  |
| 66 | Angular Gyrus R | | NC | .96 | .009 | 26.96 | .38 | **.00000031** |
|  |  | | AD | 1.02 | .007 |  |  |  |
| 67 | Precuneus L | | NC | 1.03 | .010 | 28.89 | .38 | **.00000012** |
|  |  | | AD | 1.10 | .007 |  |  |  |
| 68 | Precuneus R | | NC | 1.04 | .010 | 31.73 | .40 | **.00000003** |
|  |  | | AD | 1.11 | .008 |  |  |  |
| 69 | Paracentral Lobule L | | NC | 1.19 | .012 | .16 | .16 | .68940243 |
|  |  | | AD | 1.19 | .009 |  |  |  |
| 70 | Paracentral Lobule R | | NC | 1.12 | .011 | 2.58 | .01 | .10893285 |
|  |  | | AD | 1.15 | .008 |  |  |  |
| 71 | Caudate Nucleus L | | NC | 1.10 | .010 | .06 | .16 | .80577217 |
|  |  | | AD | 1.10 | .008 |  |  |  |
| 72 | Caudate Nucleus R | | NC | 1.06 | .010 | .23 | .20 | .63200949 |
|  |  | | AD | 1.06 | .008 |  |  |  |
| 73 | Putamen L | | NC | 1.27 | .013 | 3.87 | .10 | .04975371 |
|  |  | | AD | 1.31 | .010 |  |  |  |
| 74 | Putamen R | | NC | 1.26 | .013 | 4.98 | .14 | .02614408 |
|  |  | | AD | 1.29 | .009 |  |  |  |
| 75 | Globus Pallidus L | | NC | 1.19 | .011 | 8.54 | .25 | .00364387 |
|  |  | | AD | 1.23 | .008 |  |  |  |
| 76 | Globus Pallidus R | | NC | 1.09 | .010 | 1.86 | .05 | .17356530 |
|  |  | | AD | 1.11 | .007 |  |  |  |
| 77 | Thalamus L | | NC | 1.25 | .012 | 1.19 | .39 | .27658720 |
|  |  | | AD | 1.23 | .009 |  |  |  |
| 78 | Thalamus R | | NC | 1.22 | .012 | .74 | .32 | .38941540 |
|  |  | | AD | 1.20 | .009 |  |  |  |
| 79 | Transverse Temporal Gyrus L | | NC | 1.07 | .010 | 4.47 | .10 | .03496709 |
|  |  | | AD | 1.10 | .007 |  |  |  |
| 80 | Transverse Temporal Gyrus R | | NC | 1.07 | .010 | 2.85 | .05 | .09215691 |
|  |  | | AD | 1.09 | .007 |  |  |  |
| 81 | Superior Temporal Gyrus L | | NC | 1.02 | .010 | 16.02 | .32 | **.00007287** |
|  |  | | AD | 1.07 | .007 |  |  |  |
| 82 | Superior Temporal Gyrus R | | NC | 1.00 | .010 | 21.78 | .40 | **.00000402** |
|  |  | | AD | 1.06 | .007 |  |  |  |
| 83 | Superior Temporal Pole L | | NC | .88 | .008 | 6.75 | .15 | .00970352 |
|  |  | | AD | .91 | .006 |  |  |  |
| 84 | Superior Temporal Pole R | | NC | .92 | .008 | 4.54 | .11 | .03373339 |
|  |  | | AD | .94 | .006 |  |  |  |
| 85 | Middle Temporal Gyrus L | | NC | .93 | .008 | 19.44 | .36 | **.00001294** |
|  |  | | AD | .98 | .006 |  |  |  |
| 86 | Middle Temporal Gyrus R | | NC | .95 | .009 | 15.29 | .30 | **.00010608** |
|  |  | | AD | .99 | .006 |  |  |  |
| 87 | Middle Temporal Pole L | | NC | .90 | .008 | 4.31 | .08 | .03855768 |
|  |  | | AD | .92 | .006 |  |  |  |
| 88 | Middle Temporal Pole R | | NC | .92 | .008 | 3.78 | .08 | .05249867 |
|  |  | | AD | .94 | .006 |  |  |  |
| 89 | Inferior Temporal Gyrus L | | NC | .94 | .008 | 7.88 | .15 | .00520603 |
|  |  | | AD | .97 | .006 |  |  |  |
| 90 | Inferior Temporal Gyrus R | | NC | .96 | .008 | 5.76 | .12 | .01682444 |
|  |  | | AD | .98 | .006 |  |  |  |
|  |  | |  |  |  |  |  |  |

Abbreviations: AAL, Automated Anatomical Labeling; *EMM*, Estimated Marginal Means; *SE*, Standard Error; *d*, Cohen’s d effect size; NC, Normal Cognition; AD, Alzheimer’s Disease; Data adjusted for age and sex; *p* <.0005.

**Supplementary table 2.** **Table of mean T1-w/T2-w ratio values for all AAL brain regions compared between AD and NC subjects without image bias correction.** Areas showing significantly higher T1-w/T2-w ratios in AD compared to CN subjects are displayed in bold.

|  |  |  | No Bias Correction | | |  |  |
| --- | --- | --- | --- | --- | --- | --- | --- |
| **AAL** | **Region** | **Diagnosis** | ***EMM*** | ***SE*** | ***F*** | ***d*** | **P-value** |
| 1 | Precentral Gyrus L | NC | .94 | .008 | 2.02 | .04 | .155020929 |
|  |  | AD | .95 | .006 |  |  |  |
| 2 | Precentral Gyrus R | NC | .97 | .009 | 1.40 | .01 | .237211492 |
|  |  | AD | .99 | .007 |  |  |  |
| 3 | Superior Frontal Gyrus L | NC | .86 | .008 | 5.84 | .03 | .016018675 |
|  |  | AD | .89 | .006 |  |  |  |
| 4 | Superior Frontal Gyrus R | NC | .87 | .008 | 5.55 | .01 | .018907383 |
|  |  | AD | .89 | .006 |  |  |  |
| 5 | Superior Orbital Frontal Gyrus L | NC | .87 | .007 | .91 | .11 | .339739884 |
|  |  | AD | .88 | .005 |  |  |  |
| 6 | Superior Orbital Frontal Gyrus R | NC | .88 | .007 | 1.89 | .06 | .169540644 |
|  |  | AD | .89 | .005 |  |  |  |
| 7 | Middle Frontal Gyrus L | NC | .83 | .007 | 3.35 | .03 | .068037093 |
|  |  | AD | .85 | .005 |  |  |  |
| 8 | Middle Frontal Gyrus R | NC | .83 | .007 | 4.41 | .03 | .036204325 |
|  |  | AD | .85 | .005 |  |  |  |
| 9 | Middle Orbital Frontal Gyrus L | NC | .85 | .007 | .03 | .16 | .860415523 |
|  |  | AD | .85 | .005 |  |  |  |
| 10 | Middle Orbital Frontal Gyrus R | NC | .85 | .007 | .21 | .13 | .649990770 |
|  |  | AD | .86 | .005 |  |  |  |
| 11 | Inferior Frontal Gyrus Pars Opercularis L | NC | .87 | .007 | 2.16 | .01 | .142825535 |
|  |  | AD | .88 | .006 |  |  |  |
| 12 | Inferior Frontal Gyrus Pars Opercularis R | NC | .86 | .008 | 5.08 | .09 | .024649034 |
|  |  | AD | .89 | .006 |  |  |  |
| 13 | Inferior Frontal Gyrus Pars Triangularis L | NC | .84 | .007 | .27 | .12 | .606964369 |
|  |  | AD | .84 | .005 |  |  |  |
| 14 | Inferior Frontal Gyrus Pars Triangularis R | NC | .86 | .007 | 1.43 | .01 | .232716288 |
|  |  | AD | .87 | .005 |  |  |  |
| 15 | Inferior Frontal Gyrus Pars Orbitalis L | NC | .90 | .008 | .36 | .10 | .550841090 |
|  |  | AD | .90 | .006 |  |  |  |
| 16 | Inferior Frontal Gyrus Pars Orbitalis R | NC | .94 | .008 | .64 | .05 | .423350945 |
|  |  | AD | .95 | .006 |  |  |  |
| 17 | Rolandic Operculum L | NC | .95 | .009 | 2.28 | .01 | .131725687 |
|  |  | AD | .97 | .007 |  |  |  |
| 18 | Rolandic Operculum R | NC | .92 | .009 | 7.82 | .14 | .005389764 |
|  |  | AD | .95 | .007 |  |  |  |
| 19 | Supplementary Motor Area L | NC | .86 | .009 | 3.16 | .02 | .076297309 |
|  |  | AD | .88 | .007 |  |  |  |
| 20 | Supplementary Motor Area R | NC | .87 | .009 | 4.42 | .02 | .036037633 |
|  |  | AD | .89 | .007 |  |  |  |
| 21 | Olfactory Cortex L | NC | .92 | .008 | 1.98 | .04 | .160282515 |
|  |  | AD | .93 | .006 |  |  |  |
| 22 | Olfactory Cortex R | NC | .91 | .008 | 2.86 | .01 | .091311768 |
|  |  | AD | .93 | .006 |  |  |  |
| 23 | Medial Frontal Gyrus L | NC | .85 | .008 | 1.69 | .13 | .194132696 |
|  |  | AD | .87 | .006 |  |  |  |
| 24 | Medial Frontal Gyrus R | NC | .85 | .008 | 1.81 | .14 | .178954335 |
|  |  | AD | .86 | .006 |  |  |  |
| 25 | Medial Orbitofrontal Cortex L | NC | .84 | .008 | .49 | .12 | .483022368 |
|  |  | AD | .85 | .006 |  |  |  |
| 26 | Medial Orbitofrontal Cortex R | NC | .83 | .007 | .92 | .10 | .339125292 |
|  |  | AD | .84 | .005 |  |  |  |
| 27 | Gyrus Rectus L | NC | .85 | .008 | 1.62 | .03 | .204172970 |
|  |  | AD | .87 | .006 |  |  |  |
| 28 | Gyrus Rectus R | NC | .87 | .008 | 2.22 | .01 | .136696899 |
|  |  | AD | .88 | .006 |  |  |  |
| 29 | Insula L | NC | .83 | .008 | 4.20 | .09 | .041002967 |
|  |  | AD | .85 | .006 |  |  |  |
| 30 | Insula R | NC | .87 | .009 | 8.73 | .20 | .003289108 |
|  |  | AD | .91 | .006 |  |  |  |
| 31 | Anterior Cingulate Gyrus L | NC | .84 | .008 | 2.69 | .02 | .101558857 |
|  |  | AD | .86 | .006 |  |  |  |
| 32 | Anterior Cingulate Gyrus R | NC | .83 | .008 | 5.66 | .07 | .017780789 |
|  |  | AD | .86 | .006 |  |  |  |
| 33 | Midcingulate Area L | NC | .84 | .009 | 3.94 | .15 | .047891410 |
|  |  | AD | .86 | .006 |  |  |  |
| 34 | Midcingulate Area R | NC | .82 | .008 | 7.09 | .22 | .008004431 |
|  |  | AD | .85 | .006 |  |  |  |
| 35 | Posterior Cingulate Cyrus L | NC | 1.10 | .011 | 5.97 | .16 | .014947769 |
|  |  | AD | 1.14 | .008 |  |  |  |
| 36 | Posterior Cingulate Cyrus R | NC | 1.12 | .010 | .69 | .05 | .407780965 |
|  |  | AD | 1.13 | .008 |  |  |  |
| 37 | Hippocampus L | NC | .96 | .009 | 2.35 | .35 | .125895903 |
|  |  | AD | .94 | .006 |  |  |  |
| 38 | Hippocampus R | NC | 1.01 | .009 | 3.10 | .38 | .079196357 |
|  |  | AD | .99 | .007 |  |  |  |
| 39 | Parahippocampal Gyrus L | NC | 1.01 | .009 | 1.91 | .03 | .167365500 |
|  |  | AD | 1.03 | .007 |  |  |  |
| 40 | Parahippocampal Gyrus R | NC | 1.03 | .009 | 1.68 | .04 | .195849941 |
|  |  | AD | 1.05 | .007 |  |  |  |
| 41 | Amygdala L | NC | 1.01 | .009 | 1.59 | .06 | .207938092 |
|  |  | AD | 1.02 | .007 |  |  |  |
| 42 | Amygdala R | NC | 1.02 | .009 | 2.51 | .01 | .113625731 |
|  |  | AD | 1.04 | .007 |  |  |  |
| 43 | Calcarine Sulcus L | NC | 1.18 | .011 | 4.65 | .08 | .031648162 |
|  |  | AD | 1.21 | .008 |  |  |  |
| 44 | Calcarine Sulcus R | NC | 1.22 | .012 | 1.26 | .06 | .262891441 |
|  |  | AD | 1.24 | .009 |  |  |  |
| 45 | Cuneus L | NC | 1.10 | .011 | 3.57 | .07 | .059371934 |
|  |  | AD | 1.13 | .008 |  |  |  |
| 46 | Cuneus R | NC | 1.14 | .012 | .98 | .06 | .323573151 |
|  |  | AD | 1.16 | .009 |  |  |  |
| 47 | Lingual Gyrus L | NC | 1.19 | .011 | 3.14 | .05 | .077120592 |
|  |  | AD | 1.22 | .008 |  |  |  |
| 48 | Lingual Gyrus R | NC | 1.24 | .012 | .29 | .13 | .592919523 |
|  |  | AD | 1.25 | .009 |  |  |  |
| 49 | Superior Occipital Gyrus L | NC | 1.08 | .011 | 2.06 | .01 | .151759387 |
|  |  | AD | 1.10 | .008 |  |  |  |
| 50 | Superior Occipital Gyrus R | NC | 1.10 | .012 | .04 | .14 | .842129890 |
|  |  | AD | 1.10 | .009 |  |  |  |
| 51 | Middle Occipital Gyrus L | NC | 1.03 | .010 | 3.01 | .02 | .083555996 |
|  |  | AD | 1.05 | .008 |  |  |  |
| 52 | Middle Occipital Gyrus R | NC | 1.03 | .010 | .01 | .19 | .915198686 |
|  |  | AD | 1.03 | .008 |  |  |  |
| 53 | Inferior Occipital Cortex L | NC | 1.06 | .009 | 2.60 | .05 | .107304848 |
|  |  | AD | 1.08 | .007 |  |  |  |
| 54 | Inferior Occipital Cortex R | NC | 1.10 | .010 | .05 | .29 | .826979009 |
|  |  | AD | 1.10 | .007 |  |  |  |
| 55 | Fusiform Gyrus L | NC | 1.01 | .010 | 6.37 | .08 | .011977785 |
|  |  | AD | 1.04 | .007 |  |  |  |
| 56 | Fusiform Gyrus R | NC | 1.04 | .010 | 2.58 | .06 | .108746931 |
|  |  | AD | 1.06 | .007 |  |  |  |
| 57 | Postcentral Gyrus L | NC | .97 | .009 | 4.13 | .09 | .042811883 |
|  |  | AD | .99 | .007 |  |  |  |
| 58 | Postcentral Gyrus R | NC | .99 | .010 | 6.28 | .16 | .012566381 |
|  |  | AD | 1.03 | .007 |  |  |  |
| 59 | Superior Parietal Lobule L | NC | 1.05 | .011 | 12.24 | .23 | **.000513297** |
|  |  | AD | 1.10 | .008 |  |  |  |
| 60 | Superior Parietal Lobule R | NC | 1.06 | .011 | 7.38 | .16 | .006827863 |
|  |  | AD | 1.10 | .008 |  |  |  |
| 61 | Inferior Parietal Lobule L | NC | 1.00 | .010 | 16.17 | .29 | **.000067739** |
|  |  | AD | 1.05 | .008 |  |  |  |
| 62 | Inferior Parietal Lobule R | NC | 1.02 | .010 | 17.09 | .30 | **.000042324** |
|  |  | AD | 1.07 | .008 |  |  |  |
| 63 | Supramarginal Gyrus L | NC | .92 | .009 | 8.01 | .16 | .004849522 |
|  |  | AD | .95 | .007 |  |  |  |
| 64 | Supramarginal Gyrus R | NC | .93 | .009 | 13.01 | .25 | **.000343865** |
|  |  | AD | .97 | .007 |  |  |  |
| 65 | Angular Gyrus L | NC | .97 | .010 | 13.056 | .23 | **.000335760** |
|  |  | AD | 1.02 | .007 |  |  |  |
| 66 | Angular Gyrus R | NC | 1.00 | .010 | 6.05 | .10 | .014254830 |
|  |  | AD | 1.03 | .008 |  |  |  |
| 67 | Precuneus L | NC | 1.05 | .011 | 15.96 | .29 | **.000075372** |
|  |  | AD | 1.11 | .008 |  |  |  |
| 68 | Precuneus R | NC | 1.08 | .012 | 15.30 | .27 | **.000105657** |
|  |  | AD | 1.14 | .009 |  |  |  |
| 69 | Paracentral Lobule L | NC | 1.08 | .014 | .30 | .09 | .586200770 |
|  |  | AD | 1.09 | .010 |  |  |  |
| 70 | Paracentral Lobule R | NC | 1.05 | .012 | 2.16 | .02 | .142390214 |
|  |  | AD | 1.07 | .009 |  |  |  |
| 71 | Caudate Nucleus L | NC | 1.13 | .011 | 3.28 | .35 | .070823615 |
|  |  | AD | 1.10 | .008 |  |  |  |
| 72 | Caudate Nucleus R | NC | 1.11 | .011 | 2.16 | .31 | .142148917 |
|  |  | AD | 1.09 | .008 |  |  |  |
| 73 | Putamen L | NC | 1.32 | .014 | .40 | .07 | .525618173 |
|  |  | AD | 1.34 | .010 |  |  |  |
| 74 | Putamen R | NC | 1.35 | .014 | 3.00 | .08 | .084216443 |
|  |  | AD | 1.38 | .010 |  |  |  |
| 75 | Globus Pallidus L | NC | 1.26 | .012 | 3.75 | .13 | .053482068 |
|  |  | AD | 1.29 | .009 |  |  |  |
| 76 | Globus Pallidus R | NC | 1.15 | .011 | .10 | .05 | .755338032 |
|  |  | AD | 1.16 | .008 |  |  |  |
| 77 | Thalamus L | NC | 1.44 | .014 | 1.17 | .33 | .280593715 |
|  |  | AD | 1.42 | .010 |  |  |  |
| 78 | Thalamus R | NC | 1.43 | .014 | .35 | .24 | .553355306 |
|  |  | AD | 1.42 | .010 |  |  |  |
| 79 | Transverse Temporal Gyrus L | NC | 1.07 | .010 | 1.01 | .03 | .315234797 |
|  |  | AD | 1.09 | .008 |  |  |  |
| 80 | Transverse Temporal Gyrus R | NC | 1.07 | .010 | 1.82 | .01 | .177798976 |
|  |  | AD | 1.09 | .007 |  |  |  |
| 81 | Superior Temporal Gyrus L | NC | .98 | .009 | 6.52 | .15 | .010981050 |
|  |  | AD | 1.01 | .007 |  |  |  |
| 82 | Superior Temporal Gyrus R | NC | .96 | .009 | 18.01 | .30 | **.000026550** |
|  |  | AD | 1.01 | .007 |  |  |  |
| 83 | Superior Temporal Pole L | NC | .80 | .007 | 4.26 | .06 | .039648894 |
|  |  | AD | .82 | .005 |  |  |  |
| 84 | Superior Temporal Pole R | NC | .84 | .008 | 5.07 | .10 | .024860289 |
|  |  | AD | .87 | .006 |  |  |  |
| 85 | Middle Temporal Gyrus L | NC | .89 | .008 | 11.56 | .22 | .000731090 |
|  |  | AD | .93 | .006 |  |  |  |
| 86 | Middle Temporal Gyrus R | NC | .93 | .009 | 8.71 | .12 | .003328057 |
|  |  | AD | .96 | .007 |  |  |  |
| 87 | Middle Temporal Pole L | NC | .78 | .007 | 9.28 | .17 | .002452176 |
|  |  | AD | .81 | .005 |  |  |  |
| 88 | Middle Temporal Pole R | NC | .80 | .007 | 11.87 | .23 | .000621285 |
|  |  | AD | .83 | .005 |  |  |  |
| 89 | Inferior Temporal Gyrus L | NC | .88 | .007 | 11.14 | .14 | **.000915779** |
|  |  | AD | .91 | .006 |  |  |  |
| 90 | Inferior Temporal Gyrus R | NC | .92 | .008 | 9.36 | .07 | .002344465 |
|  |  | AD | .952 | .006 |  |  |  |

Abbreviations: AAL, Automated Anatomical Labeling; *EMM*, Estimated Marginal Means; *SE*, Standard Error; *d*, Cohen’s d effect size; NC, Normal Cognition; AD, Alzheimer’s Disease; Data adjusted for age and sex; *p* <.0005.

**Supplementary table 3.** **Table of mean T1-w/T2-w ratio values for all AAL brain regions compared between AD and NC subjects using age and sex matched individuals (n=50).** Areas showing significantly higher T1-w/T2-w ratios in AD compared to CN subjects are displayed in bold.

|  |  |  | Age and sex matched (n=50) | | | | |
| --- | --- | --- | --- | --- | --- | --- | --- |
| **AAL** | **Region** | **Diagnosis** | ***EMM*** | ***SE*** | ***F*** | ***d*** | **P-value** |
| 1 | Precentral Gyrus L | NC | 1.09 | .021 | 2.81 | .47 | .100531524 |
|  |  | AD | 1.14 | .021 |  |  |  |
| 2 | Precentral Gyrus R | NC | 1.12 | .021 | 3.56 | .52 | .065486786 |
|  |  | AD | 1.18 | .021 |  |  |  |
| 3 | Superior Frontal Gyrus L | NC | 1.02 | .020 | 5.33 | .66 | .025504140 |
|  |  | AD | 1.08 | .020 |  |  |  |
| 4 | Superior Frontal Gyrus R | NC | 1.03 | .020 | 5.59 | .67 | .022301262 |
|  |  | AD | 1.09 | .020 |  |  |  |
| 5 | Superior Orbital Frontal Gyrus L | NC | .99 | .017 | 4.19 | .57 | .046317027 |
|  |  | AD | 1.04 | .017 |  |  |  |
| 6 | Superior Orbital Frontal Gyrus R | NC | 1.00 | .018 | 2.77 | .46 | .103069720 |
|  |  | AD | 1.04 | .018 |  |  |  |
| 7 | Middle Frontal Gyrus L | NC | .99 | .020 | 5.37 | .67 | .024989283 |
|  |  | AD | 1.05 | .020 |  |  |  |
| 8 | Middle Frontal Gyrus R | NC | 1.00 | .019 | 6.26 | .71 | .015945832 |
|  |  | AD | 1.07 | .019 |  |  |  |
| 9 | Middle Orbital Frontal Gyrus L | NC | .96 | .016 | 5.54 | .66 | .022958444 |
|  |  | AD | 1.01 | .016 |  |  |  |
| 10 | Middle Orbital Frontal Gyrus R | NC | .97 | .016 | 5.25 | .63 | .026597171 |
|  |  | AD | 1.02 | .016 |  |  |  |
| 11 | Inferior Frontal Gyrus Pars Opercularis L | NC | .98 | .018 | 6.72 | .73 | .012754116 |
|  |  | AD | 1.05 | .018 |  |  |  |
| 12 | Inferior Frontal Gyrus Pars Opercularis R | NC | .98 | .018 | 8.20 | .81 | .006293423 |
|  |  | AD | 1.05 | .018 |  |  |  |
| 13 | Inferior Frontal Gyrus Pars Triangularis L | NC | .97 | .019 | 6.27 | .71 | .015906473 |
|  |  | AD | 1.04 | .019 |  |  |  |
| 14 | Inferior Frontal Gyrus Pars Triangularis R | NC | .98 | .018 | 6.29 | .68 | .015767251 |
|  |  | AD | 1.05 | .018 |  |  |  |
| 15 | Inferior Frontal Gyrus Pars Orbitalis L | NC | 1.00 | .018 | 4.24 | .58 | .045083812 |
|  |  | AD | 1.05 | .018 |  |  |  |
| 16 | Inferior Frontal Gyrus Pars Orbitalis R | NC | 1.03 | .018 | 3.98 | .55 | .052029706 |
|  |  | AD | 1.08 | .018 |  |  |  |
| 17 | Rolandic Operculum L | NC | 1.00 | .018 | 4.96 | .62 | .030863663 |
|  |  | AD | 1.06 | .018 |  |  |  |
| 18 | Rolandic Operculum R | NC | .97 | .019 | 5.98 | .68 | .018324796 |
|  |  | AD | 1.04 | .019 |  |  |  |
| 19 | Supplementary Motor Area L | NC | 1.03 | .021 | 3.93 | .55 | .053407176 |
|  |  | AD | 1.09 | .021 |  |  |  |
| 20 | Supplementary Motor Area R | NC | 1.03 | .020 | 4.92 | .62 | .031560015 |
|  |  | AD | 1.10 | .020 |  |  |  |
| 21 | Olfactory Cortex L | NC | .95 | .016 | 2.59 | .44 | .114420632 |
|  |  | AD | .98 | .016 |  |  |  |
| 22 | Olfactory Cortex R | NC | .93 | .017 | 4.42 | .59 | .041097325 |
|  |  | AD | .98 | .017 |  |  |  |
| 23 | Medial Frontal Gyrus L | NC | .97 | .019 | 5.35 | .66 | .025261761 |
|  |  | AD | 1.03 | .019 |  |  |  |
| 24 | Medial Frontal Gyrus R | NC | .96 | .020 | 5.23 | .65 | .026843969 |
|  |  | AD | 1.03 | .020 |  |  |  |
| 25 | Medial Orbitofrontal Cortex L | NC | .91 | .017 | 6.27 | .71 | .015898254 |
|  |  | AD | .97 | .017 |  |  |  |
| 26 | Medial Orbitofrontal Cortex R | NC | .88 | .016 | 5.68 | .67 | .021367361 |
|  |  | AD | .94 | .016 |  |  |  |
| 27 | Gyrus Rectus L | NC | .94 | .017 | 3.05 | .48 | .087678439 |
|  |  | AD | .98 | .017 |  |  |  |
| 28 | Gyrus Rectus R | NC | .93 | .018 | 3.95 | .56 | .052977696 |
|  |  | AD | .98 | .018 |  |  |  |
| 29 | Insula L | NC | .85 | .016 | 8.33 | .82 | .005926361 |
|  |  | AD | .92 | .016 |  |  |  |
| 30 | Insula R | NC | .87 | .018 | 7.68 | .76 | .008018333 |
|  |  | AD | .94 | .018 |  |  |  |
| 31 | Anterior Cingulate Gyrus L | NC | .88 | .021 | 8.05 | .81 | .006753107 |
|  |  | AD | .96 | .021 |  |  |  |
| 32 | Anterior Cingulate Gyrus R | NC | .86 | .020 | 11.21 | .95 | .001627435 |
|  |  | AD | .95 | .020 |  |  |  |
| 33 | Midcingulate Area L | NC | .92 | .018 | 8.63 | .83 | .005144401 |
|  |  | AD | .99 | .018 |  |  |  |
| 34 | Midcingulate Area R | NC | .90 | .017 | 9.22 | .85 | .003940629 |
|  |  | AD | .97 | .017 |  |  |  |
| 35 | Posterior Cingulate Cyrus L | NC | 1.04 | .019 | 11.14 | .93 | .001682431 |
|  |  | AD | 1.13 | .019 |  |  |  |
| 36 | Posterior Cingulate Cyrus R | NC | 1.05 | .017 | 3.48 | .51 | .068367528 |
|  |  | AD | 1.09 | .017 |  |  |  |
| 37 | Hippocampus L | NC | .88 | .015 | .02 | .05 | .889864582 |
|  |  | AD | .88 | .015 |  |  |  |
| 38 | Hippocampus R | NC | .91 | .015 | .16 | .12 | .692153478 |
|  |  | AD | .90 | .015 |  |  |  |
| 39 | Parahippocampal Gyrus L | NC | .98 | .018 | .55 | .20 | .463769480 |
|  |  | AD | 1.00 | .018 |  |  |  |
| 40 | Parahippocampal Gyrus R | NC | .98 | .016 | .60 | .21 | .441446363 |
|  |  | AD | 1.00 | .016 |  |  |  |
| 41 | Amygdala L | NC | .99 | .018 | 2.00 | .39 | .164484821 |
|  |  | AD | 1.02 | .018 |  |  |  |
| 42 | Amygdala R | NC | .99 | .016 | 3.42 | .50 | .070774037 |
|  |  | AD | 1.03 | .016 |  |  |  |
| 43 | Calcarine Sulcus L | NC | 1.11 | .020 | 6.06 | .68 | .017650653 |
|  |  | AD | 1.18 | .020 |  |  |  |
| 44 | Calcarine Sulcus R | NC | 1.13 | .020 | 8.44 | .78 | .005621823 |
|  |  | AD | 1.21 | .020 |  |  |  |
| 45 | Cuneus L | NC | 1.05 | .018 | 7.73 | .73 | .007856892 |
|  |  | AD | 1.12 | .018 |  |  |  |
| 46 | Cuneus R | NC | 1.09 | .018 | 5.64 | .62 | .021803137 |
|  |  | AD | 1.15 | .018 |  |  |  |
| 47 | Lingual Gyrus L | NC | 1.09 | .019 | 4.18 | .57 | .046605485 |
|  |  | AD | 1.14 | .019 |  |  |  |
| 48 | Lingual Gyrus R | NC | 1.12 | .020 | 2.45 | .44 | .124173491 |
|  |  | AD | 1.16 | .020 |  |  |  |
| 49 | Superior Occipital Gyrus L | NC | 1.04 | .017 | 3.00 | .47 | .090011250 |
|  |  | AD | 1.08 | .017 |  |  |  |
| 50 | Superior Occipital Gyrus R | NC | 1.06 | .017 | 3.05 | .46 | .087395277 |
|  |  | AD | 1.10 | .017 |  |  |  |
| 51 | Middle Occipital Gyrus L | NC | 1.00 | .016 | 7.10 | .72 | .010601337 |
|  |  | AD | 1.06 | .016 |  |  |  |
| 52 | Middle Occipital Gyrus R | NC | .99 | .015 | 5.69 | .63 | .021291197 |
|  |  | AD | 1.04 | .015 |  |  |  |
| 53 | Inferior Occipital Cortex L | NC | 1.03 | .017 | 2.44 | .42 | .125470744 |
|  |  | AD | 1.07 | .017 |  |  |  |
| 54 | Inferior Occipital Cortex R | NC | 1.05 | .017 | 1.86 | .36 | .178778637 |
|  |  | AD | 1.08 | .017 |  |  |  |
| 55 | Fusiform Gyrus L | NC | .97 | .017 | 2.76 | .47 | .103528713 |
|  |  | AD | 1.01 | .017 |  |  |  |
| 56 | Fusiform Gyrus R | NC | .97 | .017 | 2.15 | .41 | .149632568 |
|  |  | AD | 1.01 | .017 |  |  |  |
| 57 | Postcentral Gyrus L | NC | 1.10 | .020 | 3.50 | .52 | .067908752 |
|  |  | AD | 1.16 | .020 |  |  |  |
| 58 | Postcentral Gyrus R | NC | 1.09 | .019 | 7.25 | .74 | .009843773 |
|  |  | AD | 1.16 | .019 |  |  |  |
| 59 | Superior Parietal Lobule L | NC | 1.08 | .022 | 5.52 | .65 | .023151083 |
|  |  | AD | 1.15 | .022 |  |  |  |
| 60 | Superior Parietal Lobule R | NC | 1.07 | .020 | 6.18 | .69 | .016577580 |
|  |  | AD | 1.14 | .020 |  |  |  |
| 61 | Inferior Parietal Lobule L | NC | 1.05 | .021 | 10.34 | .91 | .002381669 |
|  |  | AD | 1.15 | .021 |  |  |  |
| 62 | Inferior Parietal Lobule R | NC | 1.04 | .019 | 15.11 | 1.08 | **.000324436** |
|  |  | AD | 1.14 | .019 |  |  |  |
| 63 | Supramarginal Gyrus L | NC | .96 | .018 | 9.35 | .85 | .003717938 |
|  |  | AD | 1.04 | .018 |  |  |  |
| 64 | Supramarginal Gyrus R | NC | .972 | .018 | 12.68 | .98 | .000874195 |
|  |  | AD | 1.06 | .018 |  |  |  |
| 65 | Angular Gyrus L | NC | .98 | .018 | 9.85 | .88 | .002967069 |
|  |  | AD | 1.06 | .018 |  |  |  |
| 66 | Angular Gyrus R | NC | .98 | .017 | 12.52 | .96 | .000933689 |
|  |  | AD | 1.07 | .017 |  |  |  |
| 67 | Precuneus L | NC | 1.04 | .020 | 11.65 | .93 | .001351056 |
|  |  | AD | 1.14 | .020 |  |  |  |
| 68 | Precuneus R | NC | 1.06 | .019 | 12.46 | .96 | .000955949 |
|  |  | AD | 1.15 | .019 |  |  |  |
| 69 | Paracentral Lobule L | NC | 1.18 | .023 | 3.03 | .43 | .088668724 |
|  |  | AD | 1.23 | .023 |  |  |  |
| 70 | Paracentral Lobule R | NC | 1.13 | .024 | 3.00 | .46 | .090096836 |
|  |  | AD | 1.19 | .024 |  |  |  |
| 71 | Caudate Nucleus L | NC | 1.10 | .020 | .42 | .17 | .519655032 |
|  |  | AD | 1.12 | .020 |  |  |  |
| 72 | Caudate Nucleus R | NC | 1.07 | .019 | .02 | .05 | .901061817 |
|  |  | AD | 1.07 | .019 |  |  |  |
| 73 | Putamen L | NC | 1.27 | .026 | 6.28 | .68 | .015776219 |
|  |  | AD | 1.37 | .026 |  |  |  |
| 74 | Putamen R | NC | 1.26 | .024 | 6.51 | .70 | .014145365 |
|  |  | AD | 1.35 | .024 |  |  |  |
| 75 | Globus Pallidus L | NC | 1.20 | .022 | 5.31 | .65 | .025743623 |
|  |  | AD | 1.27 | .022 |  |  |  |
| 76 | Globus Pallidus R | NC | 1.10 | .019 | 1.70 | .36 | .200363290 |
|  |  | AD | 1.13 | .019 |  |  |  |
| 77 | Thalamus L | NC | 1.26 | .025 | .04 | .07 | .846050600 |
|  |  | AD | 1.25 | .025 |  |  |  |
| 78 | Thalamus R | NC | 1.24 | .024 | .46 | .20 | .500641987 |
|  |  | AD | 1.22 | .024 |  |  |  |
| 79 | Transverse Temporal Gyrus L | NC | 1.05 | .020 | 6.59 | .72 | .013580909 |
|  |  | AD | 1.13 | .020 |  |  |  |
| 80 | Transverse Temporal Gyrus R | NC | 1.06 | .018 | 4.96 | .62 | .030846464 |
|  |  | AD | 1.12 | .018 |  |  |  |
| 81 | Superior Temporal Gyrus L | NC | 1.03 | .019 | 7.27 | .77 | .009766599 |
|  |  | AD | 1.10 | .019 |  |  |  |
| 82 | Superior Temporal Gyrus R | NC | 1.01 | .020 | 12.43 | .97 | .000967968 |
|  |  | AD | 1.11 | .020 |  |  |  |
| 83 | Superior Temporal Pole L | NC | .89 | .017 | 3.89 | .56 | .054621381 |
|  |  | AD | .94 | .017 |  |  |  |
| 84 | Superior Temporal Pole R | NC | .93 | .018 | 2.28 | .42 | .137545688 |
|  |  | AD | .96 | .018 |  |  |  |
| 85 | Middle Temporal Gyrus L | NC | .94 | .017 | 7.17 | .75 | .010233303 |
|  |  | AD | 1.00 | .017 |  |  |  |
| 86 | Middle Temporal Gyrus R | NC | .95 | .017 | 9.55 | .86 | .003389305 |
|  |  | AD | 1.03 | .017 |  |  |  |
| 87 | Middle Temporal Pole L | NC | .90 | .016 | 3.64 | .53 | .062842449 |
|  |  | AD | .94 | .016 |  |  |  |
| 88 | Middle Temporal Pole R | NC | .92 | .017 | 2.29 | .42 | .137450344 |
|  |  | AD | .96 | .017 |  |  |  |
| 89 | Inferior Temporal Gyrus L | NC | .95 | .015 | 4.01 | .56 | .051258928 |
|  |  | AD | .99 | .015 |  |  |  |
| 90 | Inferior Temporal Gyrus R | NC | .96 | .015 | 6.05 | .68 | .017765750 |
|  |  | AD | 1.02 | .015 |  |  |  |
|  |  |  |  |  |  |  |  |

Abbreviations: AAL, Automated Anatomical Labeling; *EMM*, Estimated Marginal Means; *SE*, Standard Error; *d*, Cohen’s d effect size; NC, Normal Cognition; AD, Alzheimer’s Disease; Data adjusted for age and sex; *p* <.0005.
